# Genomic characterization of a multidrug-resistant *Citrobacter portucalensis* isolate co-harboring *bla*_*KPC–*2_ and *bla*_*NDM–*1_ on distinct plasmids

**DOI:** 10.3389/fmicb.2025.1633493

**Published:** 2025-07-16

**Authors:** Junwei Huang, Kai Shen, Keqiang Chen, Junliang Wu, Yijun Zhu, Jingchao Shi

**Affiliations:** Department of Clinical Laboratory, Affiliated Jinhua Hospital, Zhejiang University School of Medicine (Jinhua Municipal Central Hospital), Jinhua, Zhejiang, China

**Keywords:** *Citrobacter portucalensis*, *bla*_*KPC–*2_, *bla*_*NDM–*1_, plasmid-mediated resistance, antimicrobial resistance, conjugation

## Abstract

**Background:**

*Citrobacter portucalensis* is an emerging multidrug-resistant (MDR) pathogen within the *Citrobacter* genus. Although individual occurrences of *bla*_*KPC–*2_ or *bla*_*NDM–*1_ have been sporadically reported, the coexistence of both carbapenemase genes in a single strain remains extremely rare.

**Methods:**

We performed whole-genome sequencing and conjugation assays on a bloodstream isolate of *C. portucalensis* (JH112) obtained from a critically ill patient. Plasmid structure, resistance determinants, and transferability were comprehensively analyzed using *in vitro* assays and bioinformatic pipelines.

**Results:**

JH112 exhibited an extensively drug-resistant phenotype and carried two major carbapenemase genes, *bla*_*KPC–*2_ and *bla*_*NDM–*1_, located on distinct plasmids. The *bla*_*KPC–*2_ gene resided on an IncFII(Yp)-type plasmid (∼110 kb) with a complete conjugation module and was successfully transferred to a recipient strain. This plasmid also harbored an O-antigen biosynthesis gene cluster, potentially enhancing host adaptation. In contrast, the *bla*_*NDM–*1_ gene was located on a 340 kb IncHI2/HI2A-type megaplasmid with incomplete conjugation machinery and failed to transfer under standard conditions. Both plasmids showed unique structural arrangements compared to known references. The chromosome also carried *bla*_*CMY–*49_ and *qnrB1*, contributing to broad-spectrum resistance.

**Conclusion:**

We report a rare clinical *C. portucalensis* isolate co-harboring two carbapenemase genes on genetically distinct plasmids with divergent mobility. This highlights the species’ potential role as a resistance gene reservoir and the need for enhanced molecular surveillance in both clinical and environmental settings.

## Introduction

The genus *Citrobacter*, a member of the Enterobacteriaceae family, comprises common opportunistic pathogens that are widely distributed in human intestines, animal surfaces, food products, aquatic systems, and healthcare environments (Yao et al., 2021). Although traditionally considered to have lower pathogenicity than high-risk pathogens such as *Escherichia coli* and *Klebsiella pneumoniae*, *Citrobacter* species have increasingly been implicated in infections among critically ill, immunocompromised, and long-term hospitalized patients (Lee et al., 2019; Ju et al., 2025). Notably, many isolates have shown high levels of antimicrobial resistance (AMR), particularly to β-lactam antibiotics (Liu et al., 2021). Of concern, certain *Citrobacter* strains are capable of acquiring and disseminating a broad range of resistance genes, serving as reservoirs and potential mediators for horizontal gene transfer (HGT) (Fonton et al., 2024). *Citrobacter portucalensis*, a recently redefined species within the genus, is phenotypically indistinguishable from *Citrobacter freundii* and therefore often misidentified by conventional biochemical methods (Ribeiro et al., 2017). Initially studied in the context of bacterial taxonomy, emerging reports have highlighted its potential to harbor clinically significant resistance determinants, especially carbapenemase genes, suggesting its underestimated role in the evolution of multidrug-resistance (MDR) (Sellera et al., 2023; Cao et al., 2021).

Carbapenems are considered last-line antibiotics against MDR Gram-negative bacteria. Among the carbapenemases, New Delhi metallo-beta-lactamase (NDM) and *K. pneumoniae* carbapenemase (KPC) are of particular clinical relevance. NDM belongs to the NDM family (Ambler class B), while KPC is a class A serine beta-lactamase; both confer resistance to carbapenems and most other beta-lactam agents. These resistance genes are often located on mobile genetic elements (MGEs) such as plasmids, integrons, and transposons, facilitating their rapid dissemination across different species and ecological boundaries, thus posing a major challenge to infection control (Juhas, 2015). The clinical implications of *bla*_*KPC–*2_ and *bla*_*NDM–*1_ carriage are profound. Infections caused by strains harboring either of these genes are associated with limited treatment options, higher morbidity, prolonged hospital stays, and increased mortality rates, particularly among vulnerable populations such as ICU patients and those with immunosuppression (Logan and Weinstein, 2017; Nordmann et al., 2011). Notably, co-harboring of both *bla*_*KPC–*2_ and *bla*_*NDM–*1_ within the same strain can further exacerbate therapeutic challenges by conferring resistance to most β-lactams, including ceftazidime-avibactam and meropenem-vaborbactam, which are typically effective against either enzyme alone (Papp-Wallace et al., 2020). Such dual-carbapenemase producers often require reliance on last-resort agents like tigecycline, colistin, or combination regimens with limited clinical efficacy and increased toxicity risks. Therefore, strains carrying both *bla*_*KPC–*2_ and *bla*_*NDM–*1_ represent a serious clinical threat, underscoring the need for prompt detection, genomic surveillance, and rigorous infection control measures. Previous studies have shown that *C. portucalensis* can act as a cryptic intermediate host for these resistance determinants, bridging their transmission between humans, animals, and the environment (Wang et al., 2022).

While sporadic cases of *C. portucalensis* carrying either *bla*_*NDM*_ or *bla*_*KPC*_ have been reported, isolates co-harboring both genes remain exceptionally rare. Current knowledge on their resistance mechanisms, plasmid architecture, and transfer dynamics is still limited, warranting further investigation.

In addition, *Citrobacter* species exhibit strong ecological adaptability, being not only isolated from clinical samples but also stably colonizing hospital effluents, sewage systems, aquatic environments, and the intestinal tracts of animals (Alglave et al., 2025). This cross-host and cross-environment persistence suggests a critical role in the “One Health” framework, where they may serve as reservoirs and recombination platforms for AMR genes (Castañeda-Barba et al., 2024). Several studies have demonstrated that *Citrobacter* can persist in humid ICU environments for extended periods, acting as a potential source of MDR outbreaks (Fonton et al., 2025; De Geyter et al., 2017).

In this study, we report a clinical bloodstream isolate of *C. portucalensis* (strain JH112), and conduct whole-genome sequencing and resistance gene profiling. Special emphasis is placed on the genetic characterization of two plasmids respectively harboring *bla*_*KPC–*2_ and *bla*_*NDM–*1_. Through comparative genomic and phylogenetic analyses, we explore their potential evolutionary pathways and transfer mechanisms, aiming to provide molecular insight into the emergence and dissemination of MDR *Citrobacter* strains and support evidence-based surveillance and control strategies.

## Materials and methods

### Bacterial strain isolation and species identification

The strain *C. portucalensis* JH112 was isolated in September 2023 from a blood specimen of a 82-year-old male patient with acute myocardial infarction at Jinhua Central Hospital, Zhejiang Province, China. The sample was cultured on Columbia blood agar plates at 37°C for 24 h, followed by purification. Initial identification was performed using matrix-assisted laser desorption/ionization time-of-flight mass spectrometry (MALDI-TOF/MS, bioMérieux, France), which suggested *Citrobacter braakii*. To achieve accurate species-level identification, the 16S rRNA gene was sequenced and compared using the EzBioCloud database^1^ (Chalita et al., 2024). Average Nucleotide Identity (ANI) analysis further confirmed the strain as *C. portucalensis*. In addition, ribosomal multilocus sequence typing (rMLST) was performed using the PubMLST species identification tool^2^ (Jolley et al., 2012). This method indexes variation across 53 ribosomal protein subunit genes (rps genes) and confirmed the species identity as *C. portucalensis*, with 100% match to the reference profile.

### Antimicrobial susceptibility testing and carbapenemase gene detection

Minimum inhibitory concentrations (MICs) for multiple antibiotics were determined using the broth microdilution method, including agents from the carbapenem (meropenem and ertapenem), cephalosporin (ceftazidime and cefepime), aminoglycoside, fluoroquinolone, sulfonamide, tigecycline, and colistin classes. MIC interpretations were based on the CLSI 2023 guidelines, except for tigecycline and colistin, which were interpreted according to EUCAST standards. *E. coli* ATCC 25922 was used as the quality control strain.

The classification of MDR and extensively drug-resistant (XDR) phenotypes was based on the criteria which consider only acquired resistance to antimicrobial agents. Intrinsic resistance mechanisms were excluded from the categorization (Magiorakos et al., 2012).

Carbapenemase production was initially screened using the NG-Test Carba 5 (NG Biotech), which detects KPC, NDM, IMP, VIM, and OXA-type carbapenemases. Positive results were confirmed by PCR amplification and Sanger sequencing for genotype identification.

### Conjugation assay and transfer frequency determination

Conjugation assays were performed using a liquid mating protocol. The donor strain JH112 was mixed with sodium azide-resistant *E. coli* J53 recipient cells at a 1:3 ratio and incubated statically in LB broth at 37°C for 18 h. Transconjugants were selected on LB agar plates containing sodium azide (100 μg/ml) and meropenem (2 μg/ml) to suppress the donor and select for transconjugants. Positive colonies were confirmed by antimicrobial susceptibility testing and PCR amplification of resistance genes. Conjugation frequency was calculated as the number of transconjugant colony-forming units (CFU) per donor CFU.

### Transformation assay

Plasmid DNA was extracted from *C. portucalensis* JH112, using the Qiagen Plasmid Midi Kit. The mixed plasmid preparation was electroporated into *E. coli* DH5α using standard conditions (2.5 kV, 200 Ω, 25 μF). Transformants were selected on LB agar plates supplemented with meropenem (2 μg/ml). Colonies growing on selective media were screened by PCR for *bla*_*NDM–*1_ and *bla*_*KPC–*2_ to determine which plasmid(s) had been acquired.

### Whole-genome sequencing and bioinformatics analysis

Genomic DNA was extracted using the TIANamp Bacterial DNA Kit (Tiangen Biotech, Beijing, China). Whole-genome sequencing of strain JH112 was conducted using a hybrid approach. Long-read sequencing was performed on the Oxford Nanopore MinION platform, and Illumina short-read sequencing was additionally performed for this strain to improve base accuracy. Hybrid genome assembly was carried out using Unicycler v0.4.9 (Wick et al., 2017), which integrates both long and short reads, and SPAdes v3.13.0 (Bankevich et al., 2012). Genome annotation was carried out with Prokka v1.14.6 (Seemann, 2014), and completeness was evaluated using BUSCO v4.1.2 (Simão et al., 2015). Resistance genes were annotated using the ResFinder (Florensa et al., 2022) and CARD databases (Alcock et al., 2020). Plasmid replicon types were identified with PlasmidFinder (Carattoli et al., 2014). Insertion sequences (ISs) were annotated using the ISfinder database (Siguier et al., 2006). Plasmid mobility elements, including oriT, relaxase, and type IV secretion system (T4SS) components, were detected using the oriTfinder tool (Li et al., 2018).

### Phylogenetic and MLST analysis

To determine the phylogenetic position of JH112 within the *C. portucalensis* population, all available *C. portucalensis* genome sequences were downloaded from the PubMLST database (as of December 2024) (Jolley et al., 2018). MLST was performed using the BIGSdb platform (Jolley and Maiden, 2010). Minimum spanning tree (MST) analysis based on MLST profiles was conducted with GrapeTree to evaluate the genetic relationships among different sequence types (STs). In addition, core genome alignment-based phylogenetic trees were visualized using the Interactive Tree of Life (iTOL)^3^ to determine the evolutionary clustering of JH112 among global isolates.

### Genetic context analysis and visualization

To characterize the genetic environment of *bla*_*KPC–*2_ and *bla*_*NDM–*1_, nucleotide sequences were aligned using BLASTn against representative reference plasmids in the NCBI database. Local synteny and structural variations were visualized using Easyfig v2.2.3. Circular maps of complete plasmid structures were generated using the Proksee platform^4^ to illustrate homologous regions, structural rearrangements, insertion sequences, and MGEs.

## Results

### Clinical case overview

An 82-year-old male patient with end-stage renal disease, chronic heart failure, hypertension, and type 2 diabetic nephropathy was admitted to Jinhua Central Hospital in August 2023 following acute cardiovascular decompensation. He was diagnosed with ST-segment elevation myocardial infarction and transferred to the ICU after emergency coronary intervention.

Despite empirical antimicrobial treatment with ceftriaxone, linezolid, and piperacillin/tazobactam, the patient’s condition remained critical. On hospital day 3, blood cultures yielded two Gram-negative bacilli, initially identified by MALDI-TOF MS as *C. braakii* and *Aeromonas caviae*. The *Citrobacter* isolate was later confirmed by 16S rRNA sequencing and whole-genome sequencing as *C. portucalensis*, while the *Aeromonas* isolate could not be revived for further characterization.

Antimicrobial susceptibility testing revealed that the *C. portucalensis* strain exhibited an MDR profile, including resistance to carbapenems, cephalosporins, aminoglycosides, and fluoroquinolones. The *A. caviae* isolate also showed MDR in preliminary testing. The patient’s condition deteriorated despite intensive care, and following family consent, life-sustaining therapy was withdrawn. The patient died on 5 September 2023.

### Antimicrobial susceptibility profile of *C. portucalensis* JH112

Antimicrobial susceptibility testing indicated that *C. portucalensis* JH112 exhibited an MDR phenotype. The strain showed high-level resistance to various β-lactams, aminoglycosides, quinolones, and combination agents, with MICs as follows: ceftazidime (>128 μg/ml), cefepime (>128 μg/ml), meropenem (32 μg/ml), ertapenem (128 μg/ml), gentamicin (4 μg/ml), rifampicin (>128 μg/ml), tobramycin (>128 μg/ml), and ceftazidime-avibactam (>128 μg/ml). The isolate remained susceptible only to tigecycline (0.25 μg/ml) and polymyxin B (<0.125 μg/ml) (Table 1).

**TABLE 1:** Minimum inhibitory concentration values demonstrating resistance transfer from *Citrobacter portucalensis* JH112 to *Escherichia coli* J53.

Antibiotics	JH112 (μg/ml)	JH112-J53 (μg/ml)	J53 (μg/ml)	ATCC25922 (μg/ml)
CAZ	>128	>128	0.25	0.25
FEP	>128	16	0.25	0.25
MEM	32	4	0.5	<0.125
IPM	16	4	<0.125	<0.125
ETP	128	16	<0.125	<0.125
AMK	32	4	4	2
CIP	32	<0.125	<0.125	<0.125
CZA	>128	0.25	0.25	<0.125
TGC	0.25	0.125	<0.125	<0.125
COL	<0.125	<0.125	0.25	0.25

CAZ, ceftazidime; FEP, cefepime; MEM, meropenem; IPM, imipenem; ETP, ertapenem; AMK, amikacin; CIP, ciprofloxacin; CZA, ceftazidime-avibactam; TGC, tigecycline; COL, colistin; JH112, clinical isolate of *Citrobacter portucalensis*; JH112-J53, transconjugant obtained by mating JH112 with *Escherichia coli* J53; J53, sodium azide-resistant recipient strain of *E. coli*; ATCC 25922, quality control strain of *E. coli* recommended by CLSI.

### Phylogenetic analysis

**FIGURE 1:**
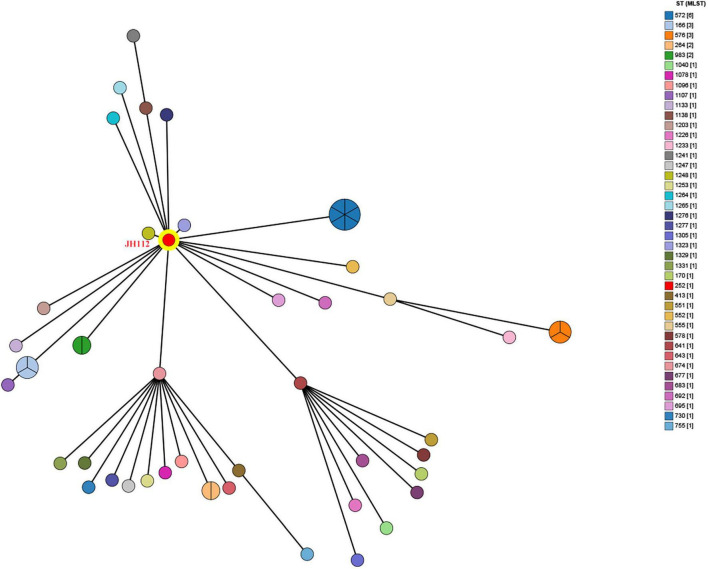
Minimum spanning tree based on multilocus sequence typing (MLST) profiles of *Citrobacter portucalensis* isolates available in the PubMLST database. Each node represents a unique sequence type (ST), with node size proportional to the number of isolates reported. The clinical isolate JH112, assigned to ST252, is highlighted in red. ST252 lies centrally and connects to multiple STs through single- or double-locus variants, indicating potential allelic proximity. However, most STs are represented by only one isolate, limiting inference of clonal expansion or dominance.

To investigate the population structure of *C. portucalensis* and determine the evolutionary placement of the clinical isolate JH112, we performed two complementary analyses. First, a MST was constructed using all STs available in the PubMLST database as of December 2024. JH112 was classified as ST252, which has only one other recorded isolate in the database. Interestingly, ST252 occupied a central node within the MST, connected to multiple other STs through single-locus variants, suggesting that it may represent an ancestral or intermediary genotype within the current population structure (Figure 1). However, due to the limited number of isolates per ST and the incomplete epidemiological metadata in the database, further inferences about its prevalence or clinical importance remain tentative.

**FIGURE 2:**
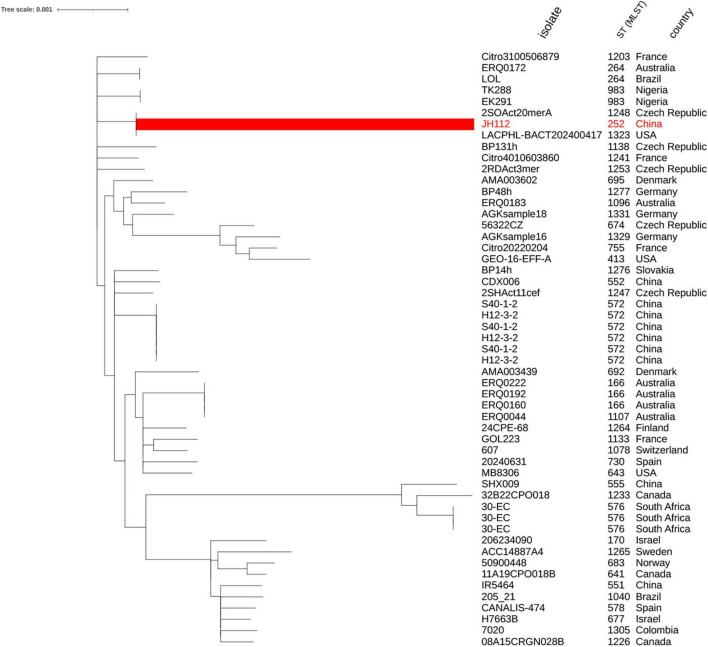
Core genome-based phylogenetic tree of *Citrobacter portucalensis* strains. The tree was constructed using concatenated alignments of core genes from *C. portucalensis* genomes with assigned STs in the PubMLST database (as of December 2024). JH112 (ST252) is highlighted in red and clusters with ST1248 and ST1323. While these STs appear phylogenetically close in the core genome tree, they remain distinct based on MLST allelic profiles and are not currently assigned to the same clonal complex. The tree shows a broadly dispersed population structure with no evidence of dominant epidemic clones, supporting the view that *C. portucalensis* is a genetically diverse species undergoing early-stage diversification.

To further explore the genomic relationship of JH112 to other *C. portucalensis* strains, we constructed a core genome-based maximum likelihood phylogenetic tree using all publicly available genomes from PubMLST. The result showed that JH112 clustered closely with environmental isolates 2SOAct20merA (Czechia) and LACPHL-BACT202400417 (United States), both of which share allelic similarities but belong to distinct STs (ST1248 and ST1323, respectively). Despite their phylogenetic proximity, these STs differ by at least one of the seven canonical MLST loci, underscoring the limitations of classical MLST in capturing fine-scale evolutionary relationships. The absence of defined clonal complexes for these STs in the PubMLST database further reflects the early diversification phase of this species. Overall, the tree supports a model of high genomic heterogeneity within *C. portucalensis*, with no evidence of dominant epidemic clones or geographic clustering, consistent with sporadic emergence from diverse ecological niches (Figure 2).

### Resistance genotype and plasmid transfer potential of JH112

Whole-genome sequencing of JH112 revealed a 4,985,931 bp chromosome (GC content: 51.6%) harboring two resistance genes: *bla*_*CMY–*49_ and *qnrB1*. *CMY-49* encodes an AmpC-type β-lactamase conferring resistance to various cephalosporins and is not inhibited by commonly used β-lactamase inhibitors. *qnrB1* encodes a pentapeptide repeat protein that protects DNA gyrase, contributing to low-level quinolone resistance.

**FIGURE 3:**
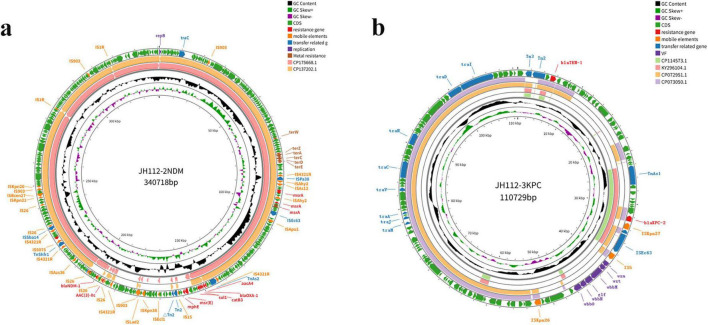
Circular maps of the resistance plasmids carried by *Citrobacter portucalensis* JH112. **(a)** pJH112-2NDM (IncHI2/IncHI2A), harboring *bla*_NDM–1_, additional resistance genes, tellurite operon, and multiple mobile elements. **(b)** pJH112-3KPC (IncFII[Yp]), containing *bla*_KPC–2_, *bla*_TEM–1_, a complete conjugation module, and an O-antigen biosynthesis cluster. Gene functions are color-coded.

Two plasmids were identified: pJH112-2NDM (IncHI2/IncHI2A, 340,718 bp) and pJH112-3KPC (IncFII(Yp), 110,729 bp). pJH112-2NDM carries multiple resistance genes, including *bla*_*NDM–*1_, two copies of *bla*_*OXA–*1_, *aac(3)-IIa*, *aac(3)-IId*, *aac(6’)-Ib3*, *msr(E)*, *mph(E)*, *catB3*, *ARR-3*, and *sul1*, forming a complex MDR island. Additionally, it harbors tellurite resistance genes (*terA*, *terC*, *terD*, *terE*, *terZ*, and *terW*), facilitating survival under heavy metal stress. The presence of MGEs such as ISAba125, IS26, ISVsa5, and ISYps3 suggests a history of recombination and potential for HGT (Table 2 and Figure 3).

**TABLE 2:** Genetic characterization of *Citrobacter portucalensis* strain JH112.

Replicon	Size (bp)	GC content (%)	Antimicrobial resistance genes	Plasmid replicon type(s)	GenBank accession
Chromosome	4,985,931	51.6	*blaCMY-49*, *qnrB1*	–	CP189867
pJH112-2NDM	340,718	47.8	*aac(3)-lla*, *aac(3)-lld*, *aac(6’)-Ib3*, *bla*_OXA–1_, *bla*_NDM–1_, *msr(E)*, *mph(E)*, *catB3*, *ARR-3*, *sul1*	IncHI2/IncHI2A	CP189868
pJH112-3KPC	110,729	52.7	*bla*_KPC–2_, *bla*_TEM–1_	IncFII(Yp)	CP189869

**FIGURE 4:**
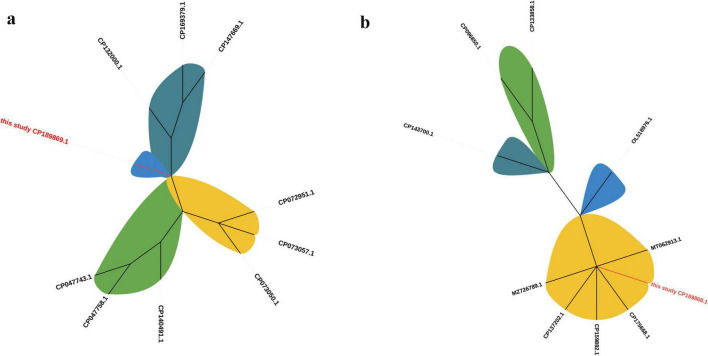
Phylogenetic trees of plasmids pJH112-2NDM and pJH112-3KPC. Maximum likelihood phylogenetic trees were constructed using complete plasmid sequences aligned with related plasmids retrieved from NCBI. **(a)** pJH112-3KPC (CP189869.1) clusters on an independent branch. **(b)** pJH112-2NDM (CP189868.1) shows high similarity to pNDM-CRNMS3 (CP175668.1) and pF3221-NDM (CP137202.1).

To further investigate the evolutionary relationships of the identified plasmids, phylogenetic analyses were performed using representative plasmid sequences retrieved from NCBI. The phylogenetic tree based on the complete plasmid sequences revealed that pJH112-3KPC formed a distinct lineage, separate from other known KPC-2–bearing plasmids, supporting its unique structural configuration and evolutionary divergence. In contrast, pJH112-2NDM clustered closely with pNDM-CRNMS3 and pF3221-NDM, indicating shared ancestry and potential derivation from a common IncHI2 backbone. These findings are consistent with the >90% sequence identity observed in pairwise alignment and highlight differing evolutionary pressures and mobility potential between the two plasmids (Figure 4).

The genetic context of *bla*_NDM–1_ includes an upstream ISAba125–IS26–YokD structure, forming a typical Tn125-like transposon. This structure is incomplete in reference plasmids pNDM-CRNMS3 and pF3221-NDM, indicating that pJH112-2NDM may represent an earlier acquisition state or an independently evolved module. Downstream, a gene cluster composed of *FrmB*, *FrmA*, *FrmR*, ISVsa5, *bla*_OXA–1_, IS26, and ISYps3 was identified, with potential mobility mediated by IS26 and ISYps3. Comparative analysis showed structural variations compared to reference plasmids, suggesting divergent evolutionary paths.

Together, these findings indicate that *bla*_NDM–1_ is embedded within a complex and modular resistance island resembling a Tn125-like transposon, with extensive flanking insertion sequences (ISAba125, IS26, ISVsa5, and ISYps3) that may enhance environmental recombination and HGT, especially under antibiotic or heavy metal selective pressure. This structure differs substantially from the *bla*_KPC–2_ genetic context, reflecting divergent mobility mechanisms.

pJH112-3KPC harbors *bla*_KPC–2_ and *bla*_TEM–1_, and was the only plasmid successfully transferred in conjugation experiments. It contains a complete conjugation module, including oriT, relaxase, and a fully functional T4SS. Conjugation assays confirmed successful transfer to *E. coli* J53, and the resulting transconjugants remained susceptible to ceftazidime-avibactam, indicating that the *bla*_NDM–1_-carrying plasmid was not co-transferred. PCR confirmed the transfer of *bla*_KPC–2_ only. The conjugation frequency of pJH112-3KPC was estimated at approximately 3.6 × 10^–3^ transconjugants per donor cell, suggesting an efficient horizontal transfer potential under laboratory conditions.

**FIGURE 5:**
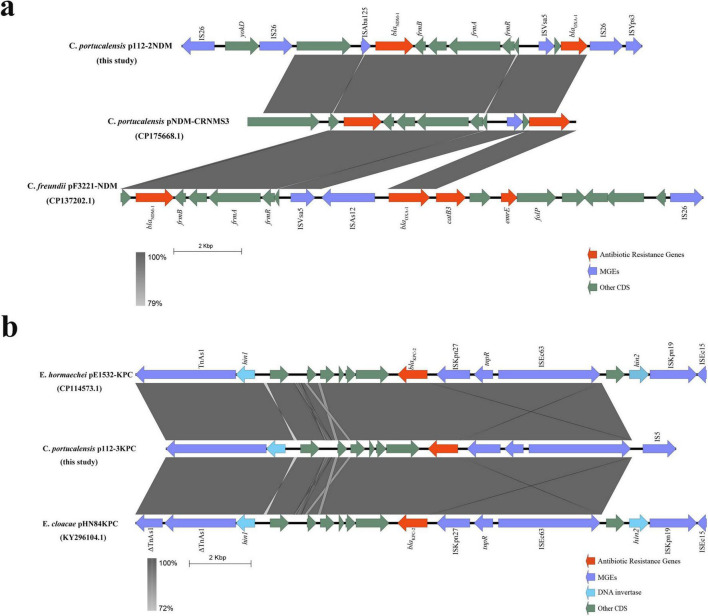
Genetic environment of *bla*_NDM–1_ and *bla*_KPC–2_ in plasmids from *Citrobacter portucalensis* JH112. **(a)** Comparative analysis of *bla*_NDM–1_ region on pJH112-2NDM showing a Tn125-like element and a downstream resistance island, relative to reference plasmids. **(b)** Structure of *bla*_KPC–2_ region on pJH112-3KPC, highlighting IS5-mediated rearrangements and a conserved downstream transposition unit. Homologous regions are shaded.

BLAST analysis revealed that no known plasmid shares complete structural identity with pJH112-3KPC, though partial homology (up to 66% coverage and 99.9% nucleotide identity) was observed with plasmids from various *Enterobacteriaceae* species, indicating a potentially novel plasmid backbone. Upstream of *bla*_KPC–2_, the ISKpn27–tnpR–ISE63 module was present; however, typical accessory elements such as hin2, ISKpn19, and ISEc15 were absent and replaced by an IS5 insertion, suggesting IS5-mediated recombination. Downstream, six conserved ORFs, hin1, and TnAs1 were fully retained, indicating a structurally complete transposition region (Figure 5).

Notably, pJH112-3KPC also carries a complete O-antigen biosynthesis gene cluster, including *wzm*, *wzt*, *wbbM*, *wbbN*, *wbbO*, and *glf*, which is uncommon in Enterobacterales plasmids and may have originated from Klebsiella species. While not classified as traditional virulence factors, these genes are involved in lipopolysaccharide biosynthesis and may enhance immune evasion and environmental fitness, suggesting that the plasmid confers both AMR and potential virulence traits.

In contrast to pJH112-2NDM, the *bla*_KPC–2_ locus is integrated within a compact and structurally complete transposition region with functional conjugation machinery, supporting high transferability. The absence of typical accessory elements and the presence of IS5 insertion suggest independent evolution and recombination events. This plasmid thus represents a distinct mobile platform combining resistance and potential virulence features.

Although oriT and relaxase elements were present on pJH112-2NDM, no transconjugants were obtained under standard laboratory conditions, suggesting impaired or inactive conjugation functionality under the tested parameters. To further evaluate its mobility potential, three independent electroporation assays were conducted using purified plasmid DNA from JH112 and *E. coli* DH5α as the recipient. Despite selection on meropenem-containing media, no transformants harboring *bla*_NDM–1_ were recovered, while a small number of colonies carrying *bla*_KPC–2_ were detected, indicating that only pJH112-3KPC was successfully introduced. These results further support that pJH112-2NDM was non-transmissible under the tested conditions, likely reflecting impaired conjugation function.

### Discussion

In recent years, the prevalence of carbapenem-resistant *Citrobacter* species has shown a notable upward trend globally, with increasing reports from Europe, the United States, and several Asian countries including China, India, and South Africa (Nobrega et al., 2023; Sommer et al., 2024). The dissemination of resistance genes such as *bla*_NDM_ and *bla*_VIM_ has been frequently associated with these isolates. *Citrobacter* spp. are commonly found in humid hospital environments, sewage systems, and ICU drainage networks. Their exceptional environmental persistence enables them to serve as difficult-to-eradicate reservoirs of AMR genes and act as critical mediators of HGT (Sellera et al., 2022).

This study reports a clinical bloodstream isolate, *C. portucalensis* JH112, recovered from a patient with multiple comorbidities, including end-stage renal disease, hypertension, diabetes mellitus, and coronary artery disease. Notably, a MDR *A. caviae* was co-isolated from the same patient, although subsequent recovery failed. The coexistence of multiple MDR pathogens under such critical infection settings may facilitate interspecies gene transfer and the acceleration of resistance evolution through community-level gene exchange. This observation is consistent with previous studies linking *Citrobacter* bacteremia with polymicrobial infections and chronic disease comorbidities (Hashimoto et al., 2021; Goncalves et al., 2024). Members of the genus *Aeromonas* possess strong integrative capacities and may act as collectors or transport platforms for resistance genes, promoting the assembly and dissemination of mobile resistance islands in collaboration with species such as *Citrobacter* (Carusi et al., 2024).

Multilocus sequence typing analysis revealed that JH112 belongs to ST252, a rarely reported ST. Although *C. portucalensis* exhibits high ST diversity, most isolates display sporadic distribution, with no dominant epidemic clones identified to date, unlike high-risk lineages such as ST11 or ST258 in *K. pneumoniae* (Li et al., 2021; Zhang et al., 2024). The presence of multiple MDR plasmids in such strains suggests that this species may currently be in a “pre-epidemic” evolutionary phase, with the potential to emerge as a dominant MDR clone under selective pressure in high-risk settings like the ICU.

A major concern highlighted in this study is the coexistence of two distinct carbapenemase-encoding plasmids within a single *C. portucalensis* isolate. From a clinical and epidemiological perspective, this configuration represents a high-risk resistance profile. The plasmid harboring *bla*_KPC–2_ demonstrated successful conjugative transfer under laboratory conditions, underscoring its potential for rapid dissemination within nosocomial environments, particularly in settings with intense antimicrobial selective pressure.

Such dual-carbapenemase producers pose a major therapeutic challenge, as they often exhibit resistance to nearly all β-lactams, including advanced β-lactam/β-lactamase inhibitor combinations like ceftazidime-avibactam and meropenem-vaborbactam, which are typically reserved for either KPC or NDM producers alone. Dual producers such as this strain are associated with poor clinical outcomes and are increasingly recognized as a significant global threat (Lee et al., 2022). This narrows available treatment options to toxic or less effective agents such as tigecycline or colistin, particularly concerning for bloodstream infections or immunocompromised patients. Consequently, infections caused by such strains are associated with increased treatment failure, prolonged hospitalization, and higher mortality.

Remarkably, one of the plasmids (pJH112-3KPC) also carried a complete O-antigen biosynthesis gene cluster, which may have been acquired from *Klebsiella* spp. Though not conventional virulence determinants, these genes contribute to lipopolysaccharide synthesis and may enhance immune evasion, colonization potential, and host adaptation (Hsieh et al., 2012). The fusion of resistance determinants with putative pathogenicity elements suggests that this plasmid may represent a hybrid resistance-adaptation platform with multifactorial advantages in clinical settings.

Structurally, pJH112-2NDM retained the ISAba125–IS26–YokD module upstream of *bla*_NDM–1_, forming a typical Tn125-like transposon not observed in comparator plasmids pNDM-CRNMS3 and pF3221-NDM. This structural configuration may reflect an earlier evolutionary acquisition event or an independent integration pathway, consistent with previous reports highlighting the role of Tn125 and ISAba125 in facilitating the global dissemination of *bla*_NDM_ (Khan et al., 2017). The downstream presence of *FrmA/B/R*, *bla*_OXA–1_, IS26, and ISYps3 forms a complex resistance locus, with Frm genes potentially enhancing bacterial stress tolerance. The *bla*_OXA–1_ gene and associated IS elements may constitute a functional composite transposon, further increasing the plasticity of the resistance region.

From a clinical and epidemiological standpoint, the carriage of multiple resistance determinants—including *aac(3)-IIa*, *aac(3)-IIb*, *aac(6’)-Ib3*, *msr(E)*, *mph(E)*, *catB3*, *ARR-3*, *sul1*—and tellurite resistance genes (*terA–terW*) greatly expands the bacterium’s potential to survive in hostile environments, such as those exposed to antibiotics or heavy metals in hospital settings. The dense array of MGEs (ISAba125, IS26, ISVsa5, and ISYps3) not only reflects a recombinogenic genome structure but also poses a risk for further HGT events across different species and ecological niches.

Although pJH112-2NDM harbors recognizable oriT and relaxase elements, no conjugative transfer was observed under standard laboratory conditions. This failure is likely multifactorial. The plasmid’s T4SS is incomplete, spanning only 9 kb and lacking auxiliary genes essential for conjugation. Its large size (>340 kb) may also impose a significant metabolic burden on the host, reducing mobilization efficiency. Furthermore, IncHI2 plasmids are known to require low-temperature induction for expression of their conjugation machinery, while our experiments were conducted at 37°C—conditions that may suppress transfer activity (Forns et al., 2005). In addition, recipient incompatibility or entry exclusion systems may contribute to the observed transfer failure. These findings align with prior research by Li et al. (2022), which reported that only 3.1% of dual-carbapenemase-producing isolates were capable of simultaneous plasmid co-transfer, emphasizing the potential role of interplasmid incompatibility and regulatory constraints. While pJH112-2NDM appears non-conjugative under current conditions, its structurally intact resistance region—rich in MGEs—suggests latent mobility potential under selective pressure or future recombination, warranting ongoing genomic surveillance and infection control vigilance.

Additionally, the chromosomal presence of *bla*_CMY–49_ and *qnrB1* suggests intrinsic resistance to β-lactams and quinolones. While CMY-type β-lactamases are typically plasmid-borne, chromosomally encoded variants, such as *bla*_CMY–37_ in *C. freundii* (Ahmed and Shimamoto, 2008) and *bla*_CMY–190_ in *Citrobacter youngae* (Liu et al., 2025), have been documented. Moreover, both *C. freundii* and *Enterobacter cloacae* are known to carry inducible chromosomal AmpC β-lactamase genes (Sader et al., 2021). Chromosomal carriage of AmpC may facilitate inducible expression under β-lactam exposure and contribute to treatment failure (Tebano et al., 2024).

Taken together, *C. portucalensis* JH112 represents a clinically relevant MDR isolate with dual-carbapenemase production, structural novelty, and potential for dissemination. This study provides a comprehensive “strain–plasmid–gene” analysis of its resistance architecture and highlights the need for enhanced surveillance of cryptic resistance reservoirs in the context of One Health.

## Conclusion

In summary, the isolation of *C. portucalensis* JH112 underscores the growing complexity and clinical relevance of carbapenem resistance within this emerging species. The strain harbors both transferable *bla*_KPC–2_ and structurally intact yet non-transmissible *bla*_NDM–1_ plasmids—representing the two most concerning carbapenemase platforms in clinical settings. The discovery of an O-antigen biosynthesis gene cluster on the *bla*_*KPC*_-plasmid suggests added potential for host adaptation and immune evasion. In addition to the two carbapenemase genes, the chromosomally encoded *bla*_CMY–49_ contributes to third-generation cephalosporin resistance and reduces the efficacy of β-lactam/β-lactamase inhibitor combinations, reflecting a multi-layered β-lactam resistance background. Furthermore, *qnrB1* provides low-level quinolone resistance, which may facilitate the selection of higher-level resistance under antibiotic pressure.

These findings not only elucidate the plasmid-level architecture of dual-carbapenemase resistance but also highlight the role of *C. portucalensis* as a potential reservoir and vector of high-risk resistance determinants in the hospital environment. Continued genomic surveillance, functional assessment of resistance plasmids, and environmental monitoring are essential to mitigate the risk of further dissemination in high-risk populations and healthcare settings.

## Data Availability

The genome sequencing data are publicly available at NCBI GenBank under the BioProject accession number: PRJNA1255133.

## Footnotes

1
https://www.ezbiocloud.net/

2
https://pubmlst.org/species-id

3
https://itol.embl.de

4
https://proksee.ca/
